# Impact of phage predation on *P. aeruginosa* adhered to human airway epithelium: major transcriptomic changes in metabolism and virulence-associated genes

**DOI:** 10.1080/15476286.2023.2216065

**Published:** 2023-05-24

**Authors:** Ana C. Brandão, Leena Putzeys, Diana P. Pires, Marleen Voet, Jan Paeshuyse, Joana Azeredo, Rob Lavigne

**Affiliations:** a Centre of Biological Engineering, University of Minho, Braga, Portugal; b LABBELS – Associate Laboratory, University of Minho, Braga, Portugal; c Laboratory of Gene Technology, KU Leuven, Leuven, Belgium; d Laboratory of Host Pathogen Interactions, KU Leuven, Leuven, Belgium

**Keywords:** *P. aeruginosa*, bacteriophage, RNA-sequencing, virulence, interactions

## Abstract

Phage therapy is a promising adjunct therapeutic approach against bacterial multidrug-resistant infections, including *Pseudomonas aeruginosa*-derived infections. Nevertheless, the current knowledge about the phage-bacteria interaction within a human environment is limited. In this work, we performed a transcriptome analysis of phage-infected *P.* *aeruginosa* adhered to a human epithelium (Nuli-1 ATCC® CRL-4011™). To this end, we performed RNA-sequencing from a complex mixture comprising phage–bacteria–human cells at early, middle, and late infection and compared it to uninfected adhered bacteria. Overall, we demonstrated that phage genome transcription is unaltered by bacterial growth and phage employs a core strategy of predation through upregulation of prophage-associated genes, a shutdown of bacterial surface receptors, and motility inhibition. In addition, specific responses were captured under lung-simulating conditions, with the expression of genes related to spermidine syntheses, sulphate acquisition, biofilm formation (both alginate and polysaccharide syntheses), lipopolysaccharide (LPS) modification, pyochelin expression, and downregulation of virulence regulators. These responses should be carefully studied in detail to better discern phage-induced changes from bacterial responses against phage. Our results establish the relevance of using complex settings that mimics *in* *vivo* conditions to study phage-bacteria interplay, being obvious the phage versatility on bacterial cell invasion.

## Introduction

*Pseudomonas aeruginosa* is an opportunistic pathogen responsible for several human infections, being a prevalent colonizer of cystic fibrosis (CF) patients’ airways [1]. *P. aeruginosa* infections are often difficult to treat due to their ability to evade host immunity and antibiotics therapy (reviewed in [2]) and, in most cases, the patient’s treatment fails. Since *P. aeruginosa* has emerged as a multi-resistance species, these bacteria represent one of the most concerning pathogens that urgently require the development of alternative therapies [3]. The increasing prevalence of multidrug-resistant (MDR) bacteria has turned the attention to phages as natural bacterial predators [4,5]. Although the phage therapy concept is over a century old, the impact of phages in bacteria and human cells is not comprehensively studied at the molecular level, leading to doubts concerning its safety and efficacy, which in turn is a major constraint towards its approval as an adjunct therapy to antibiotics treatment [4].

To better elucidate the phage-bacteria interplay at the molecular level, a number of high-throughput technologies in metagenomics, proteomics, metabolomics, and transcriptomics are now being applied in the phage research field [6–8]. Indeed, massive use of genomics/metagenomics allowed the acquisition of a large number of genomes to better understand phage distribution and evolution in ecosystems and microbiomes [9]; proteomics and metabolomics have enabled the elucidation of complex networks of proteins and metabolites from both phage and bacteria [10–15]; and transcriptomics provided a high content of information about phage-bacteria gene expression at specific stages of the phage life cycle.

Specifically, by using RNA sequencing (RNA-seq), in addition to unveiling the relevance/function of specific phage genes and interaction partners, mechanisms behind genes regulation, the delineation of transcript boundaries, the discovery of novel antisense genes and the understanding of bacterial expression circuits under phage predation are now attainable [7,8,15–24]. However, while the application of high-resolution RNA-seq-based techniques to the phage field is increasing [17,25,26], the experimental design of these works have not yet addressed the gap in capturing phage-bacteria transcriptional changes during infection situations. While some models have been applied to simulate *in vivo* lung infections (lungs from CF patients [27], CF sputum samples [28], mouse [29], and zebrafish [30] models), these have, to our knowledge, not yet been supported by phage-bacteria transcriptome analysis.

Recently, we studied the *Pseudomonas* LUZ19 phage transcriptome during *P. aeruginosa* PAO1 growth in mammalian cell culture media (MCCM) (commonly used to grow human airway epithelial cells) as a first step to evaluate growth conditions of *P. aeruginosa* phage infection process on host physiology. This exploratory study revealed the phage’s ability to efficiently progress with bacterial infection regardless of growth medium, while *P. aeruginosa* revealed major transcriptional differences at early infection related with transcriptional patterns observed in cystic fibrosis bacterial isolates, in anaerobic conditions and biofilm conditions. As phage infection progressed, the transcriptional changes decreased over time, indicating that the phage can adjust the bacterial metabolism towards an efficient infection [16].

Considering the importance of understanding bacterial responses towards phage predation and the drastic differences in bacterial behaviour under host physiology conditions, we here explore the *P. aeruginosa* transcriptome in a three-way design: phages-bacteria-human cells. For that, we applied human airway epithelial cells to better mimic phage–bacteria–host interplay, from the microbial perspective.

## Materials and methods

### Bacterial strains, phage propagation, and growth conditions

The reference strain *P. aeruginosa* PAO1 (DSM22644) from the German Collection of Microorganisms and Cell cultures was used in all experiments and was grown according to [16]. *Pseudomonas* phage LUZ19 [31] was propagated on *P. aeruginosa* PAO1 cells by standard soft agar overlay, followed by PEG8000 precipitation, and stored in phage buffer pH 7.5 (10mM Tris-HCl, 10mM MgSO_4_, 150mM NaCl) at 4°C [32]. Phage titration was performed using the double‐agar layer [33].

### Airway surface model

**Figure 1.**
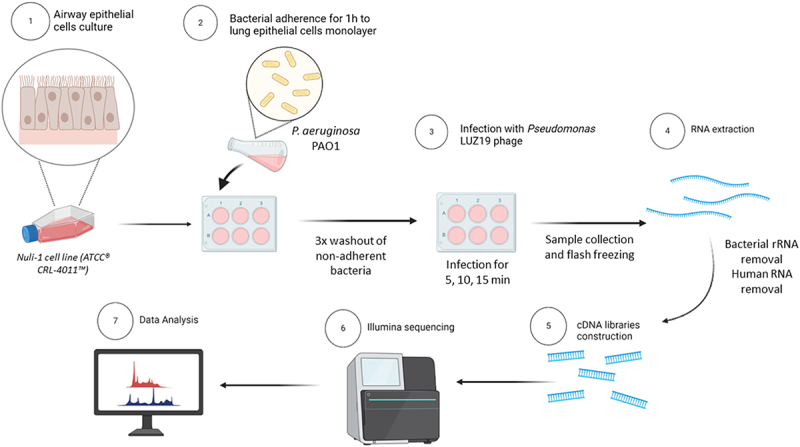
Overview of the methodology used for RNA samples acquisition from the complex mixture of Nuli-1 epithelial cells-bacteria-phage, library preparation and Illumina RNA sequencing.

The Nuli-1 cell line (ATCC® CRL-4011™) from human airway epithelium of normal genotype patient was kindly provided by Professor Brian Harvey from the Department of Molecular Medicine at the Royal College of Surgeons (Ireland). Cells were propagated on tissue culture flasks at 37°C, 5% CO_2_, and >90% humidity (Heracell 150, Heraeus) using a MCCM medium supplemented with 1% Gibco™ Antibiotic-Antimycotic (100×). When cells reached about 80% confluence, sub-culturing at a 1:3 flask ratio was performed. Next, cells were used to seed six-well tissue culture-treated plates, at 3 × 10^5^cells/mL in pre-warmed MCCM medium (without antibiotic/antimycotic), being incubated at 37°C, with 5% CO_2_, and with >90% humidity until reaching confluency. After obtaining a cell monolayer in six-well plates, these were used to perform synchronized infection assays of adhered bacteria with LUZ19 phage (Figure 1).

### Bacterial adherence to lung epithelial cells monolayer

*P. aeruginosa* PAO1 overnight cultures were centrifuged (8,000×g, 4°C, 5 min), the pellet was washed twice with Phosphate Buffered Saline (PBS: 137 mM NaCl, 10 mM Sodium Phosphate Dibasic, 2.7 mM Potassium Chloride, and Potassium Phosphate Monobasic, pH = 7.4) and the OD_600nm_ was adjusted to 0.13 (approximately 1 × 10^8^CFU/mL) with MCCM medium. Then, 1mL of bacterial suspension was added to the wells containing NuLi-1 cells, and the six-well plates were incubated at 37°C for 1h to allow bacterial cells to adhere to the Nuli-1 cell monolayer. Three independent experimental assays allowed us to quantify that at this point per six-well plate containing 2.5 × 10^5^ lung epithelial cells/mL and 3.7 × 10^7^CFU/mL of bacteria.

### Synchronized infection of adhered bacteria with LUZ19 phage

Non-adhered bacteria were removed by washing the wells with PBS and in the control plate (bacteria adhered to epithelial monolayer), adhered bacteria were collected by mechanical scraping and resuspended in 1:10 vol of an ice-cold stop solution (1:10 buffered phenol, 9:10 absolute EtOH). This sample was flash-frozen in liquid nitrogen to block RNA transcription and degradation until sample processing. In the remaining plates, after removal of non-adhered bacteria by consecutive washing with PBS, phage LUZ19 was added at high multiplicity of infection (MOI) of 75 (to ensure synchronous infection of the culture) in MCCM medium. Plates were incubated for 5, 10, and 15min at 37°C, 5% CO_2_. At each time point, the supernatant was removed quickly, and infected bacteria were collected as described in the previous section. Triplicate samples from synchronized infections and control samples (*t* = 0 min, uninfected bacteria adhered to epithelial cells monolayer) were used for total RNA extraction.

### Total RNA extraction, quantification, quality assessment, and rRNA depletion

Three independent biological assays were performed, collecting a complex mixture of *P. aeruginosa* PAO1 adhered to Nuli-1 cells and infected with LUZ19 phage for 0min (control), 5min (early-phage infection), 10min (middle-phage infection), and 15min (late-phage infection).

The samples stored at −80°C were thawed on ice and centrifuged (3,345×g, 4°C, 20min). Total RNA extraction, genomic DNA (gDNA) removal, and RNA precipitation were performed as previously described [16]. Subsequently, the concentration, purity, absence of genomic DNA, and RNA integrity were measured following methodologies described in [16].

Samples with adequate RNA integrity (RNA integrity number (RIN) > 8) were treated with MICROBEnrich^TM^ kit (Invitrogen) to deplete mammalian RNA from the complex total RNA mixtures, according to the manufacturer’s instructions.

### cDNA libraries preparation and sequencing

Mammalian RNA-depleted samples (*n* = 3 per condition) were directly used to deplete bacterial rRNA and cDNA libraries construction following Ribo-zero (Illumina) manufacturer’s recommendations. Successful rRNA depletion was verified on a Bioanalyzer using the DNA high-sensitivity kit (Agilent), and the final concentrations were determined using the Qubit 4 Fluorometer (Invitrogen).

Samples were processed immediately, according to Illumina Stranded Total RNA Prep with Ribo-Zero Plus TruSeq Stranded mRNA Library Prep recommendations. In the end, cDNA libraries were evaluated on a Bioanalyzer (Agilent) to confirm average fragments between the range of 200–300 bp, and final concentrations were measured on a Qubit. The libraries’ normalization and pooling were performed according to the MiniSeq System Denature and Dilute Libraries Guide: protocol A from Illumina. For each run, a library of three samples with equimolar amounts was combined with PhiX control (spike-in of 0.5–2%) and paired-end sequenced (2 × 75bp) on an in-house Illumina Miniseq sequencer. After the first sequencing run, specific samples were re-sequenced to increase the sequencing depth of the phage and bacterial transcriptomes and improve the statistical power to detect differentially expressed genes.

### Data analysis

The quality of the cDNA reads was analysed using FastQC (0.11.8) [34]. Adapters and poor quality sequences were trimmed using Trimmomatic (v0.39) [35]. Paired-end reads were aligned to the LUZ19 (NC_010326.1), *P. aeruginosa* PAO1 (NC_002516.2), and Human reference genomes (assembly GRCh38.p13) using the BWA-MEM aligner [36] and SAMtools (v1.9) [37] was used to process the alignment files and assess mapping quality. Next, the aligned read pairs (fragments) were assigned to the genomic features of the phage and the host using featureCounts [38]. Alignment visualization was performed using Integrative Genomics Viewer (IGV) [39].

Reads that mapped to the human genome and bacterial rRNA reads (60–80%) were removed *in silico*. Subsequently, fragment counts were first normalized by gene length and next by the number of non-ribosomal read pairs in each sample to obtain FPKM (fragments per kilo base of transcript per million mapped fragments) values. Samples variance was analysed using principal component analysis (PCA) in R. Differential gene expression analysis was performed using a negative binomial distribution test from the DESeq2 Bioconductor package in R [40] with FDR correction. An ANOVA test was performed to identify the most significantly up- or down-regulated genes. Differentially expressed genes were considered when they had both an adjusted p-value (padj) smaller than 0.05 and Log2Fold values smaller or equal than −1.5 and higher or equal than 1.5. Heat map with the normalized count values of the differentially expressed genes from all time points for each individual sample was created using the pheatmap package in R, with gene clustering.

The bacterial transcriptome during LUZ19 phage infection when adhered to human airway epithelial cells was analysed to elucidate bacterial major responses triggered by the phage under human physiological conditions. Sequencing of the RNA samples revealed that, despite the depletion of ribosomal RNA (rRNA) and mammalian RNA, a high proportion of reads mapped to the human genome and rRNA (Figure S1). The reduced sequencing depth of the bacterial transcriptome constitutes a limitation of using the triple RNA-seq technique where most of the reads map to the mammalian host cells, which can be overcome by using high-throughput sequencer systems. Considering this technical limitation, to increase the number of reads that map to the bacterial genome, we re-sequenced three samples (4_5, 7_5, 5_10, and 4_15) to achieve at least ~500,000 prokaryotic non-rRNA reads per sample, sufficient for reliable data analysis. Thus, the number of non-ribosomic reads mapped to PAO1 ranged from 462,000 to 1,459,000, and the number of reads mapped to LUZ19 phage ranged between 15,000 and 2,000,000 (Table S1).

## Results

### *Pseudomonas* LUZ19 phage transcriptome takes over the bacterial transcriptome during the 15min of infection

**Figure 2.**
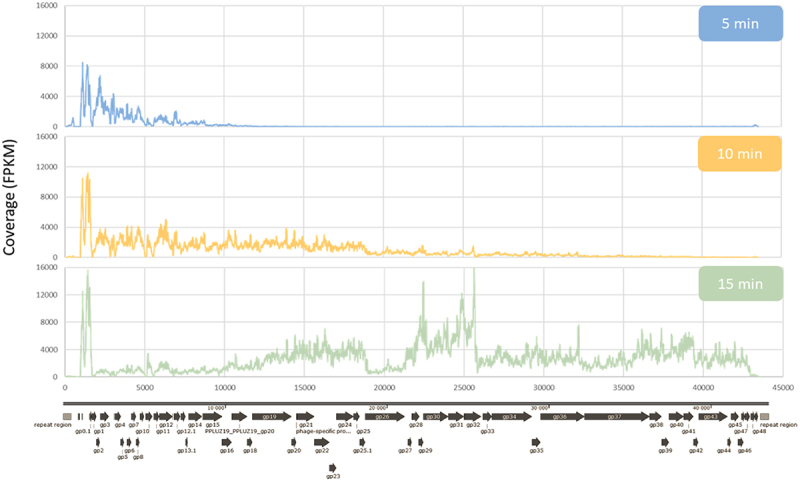
*Pseudomonas* phage LUZ19 transcriptional landscape on *P.* *aeruginosa* PAO1 adhered to Nuli-1 epithelial cells. 5min samples, 10min samples and 15min samples represent the phage transcripts at the early, middle, and late infection stage, respectively.

**Figure 3.**
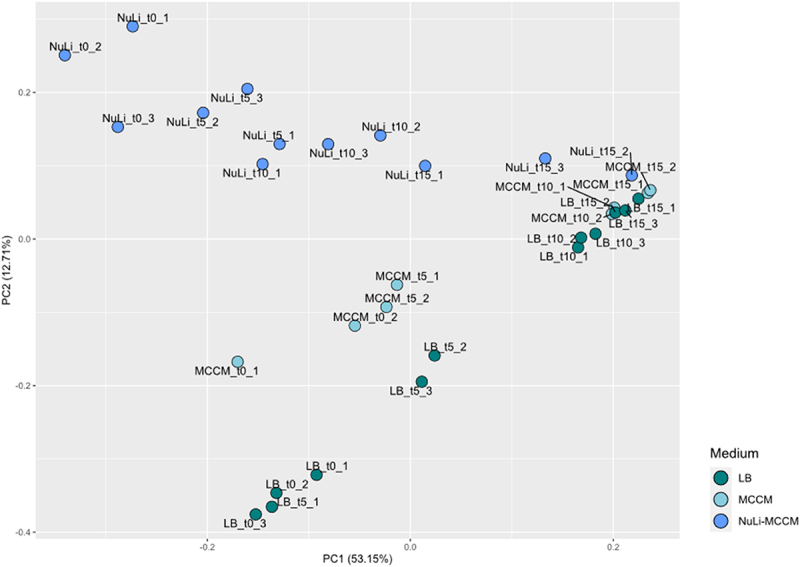
Principal component analysis of total transcriptomes from samples acquired at early (5min), middle (10min), and late infection (15min) in the presence of LB media, MCCM media, and Nuli-1 epithelial cells. The control samples (without phage presence) in each respective media are also included (t0). (Figure contains data described in (24)).

During infection of bacteria adhered to epithelial cells monolayer at 5 min post infection, reads were mapped at regions located in the start of phage genome (position 0 > ~10,000bp). Then, at 10min post infection, reads were also mapped at beginning and middle regions of phage genome (0 > ~25,000bp), while at 15min post infection a high number of reads were already mapped in regions located closer to the end of the phage genome (25,000 > 45,000bp), as the infection progresses, as visualized in Figure 2. Under these conditions, the number of reads was significantly reduced (Figure S2) as a consequence of performing triple RNA-seq. In addition, the burst size was reduced (Figure S3) because of a lower bacterial cell growth rate in these conditions. Considering similarities between phage genes expression patterns obtained in LB, MCCM [16] and in the conditions reported in this study (Figure 2), we performed a Principal Component Analysis (PCA; Figure 3) of the overall transcriptomes. Before phage exposure (*t* = 0min) bacteria have very distinct gene expression patterns when comparing the different media conditions, yet the presence of Nuli-1 epithelial cells introduces a variable that leads to a very distinct transcriptome from both MCCM medium and LB medium. Looking closer to the samples that are under phage exposure, the transcriptomes in different media begin to converge over time until the end of the infection. Overall, phage transcription mechanisms seem to proceed efficiently with minimal changes.

#### *P.* *aeruginosa* PAO1 Differential Expressed Genes (DEGs) during phage infection in the presence of Nuli-1 epithelial cells

**Figure 4.**
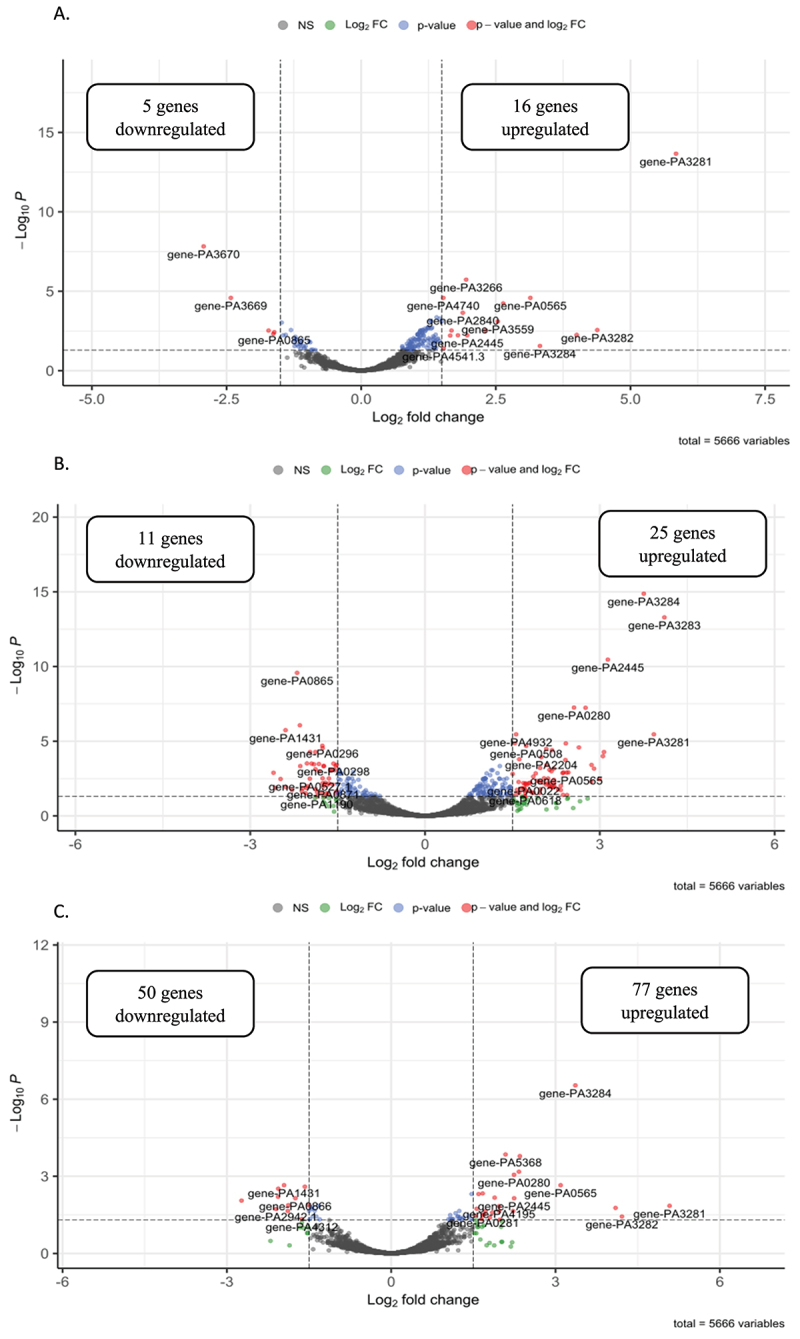
Volcano plots representing *P.* *aeruginosa* PAO1 DEGs after 5 vs. 0min (A.), 10 vs. 0min (B.), and 15 vs. 0min (C.) of infection with LUZ19 phage (Log2fold change ≥ |1.5| and padj ≤0.05).

The transcriptome of *P. aeruginosa* PAO1 in the presence of Nuli-1 epithelial cells was analysed during phage infection and compared to the transcriptome of uninfected bacteria. Thresholds used to find Differentially Expressed Genes (DEGs) were placed at Log2Fold ≥ |1.5| and padj ≤0.05. Under these conditions, 21 DEGs were obtained 5 vs. 0min (Figure 4(a)), 36 DEGs at 10 vs. 0min (Figure 4(b)), and 127 DEGs at 15 vs. 0min (Figure 4(c)), as shown in Table S2 and Figure S4. Generally, DEGs upregulated during early infection were associated with lipid A modification, glycerol metabolism, translation (tRNA biosynthesis, decrease in hibernating ribosome dimers), the prokaryotic degradome, and phosphate acquisition, whereas aromatic amino acids transport DEGs were downregulated. At 10 vs. 0min, genes involved in sulphate transport, phosphate transport, spermidine biosynthesis, glycine metabolism, pyochelin biosynthesis, and protein folding are upregulated. By contrast, the aromatic amino acids transport, arginine/ornithine transport, lipid A modification, lactate metabolism, and several transcriptional regulators are relatively downregulated at 10 vs. 0min post infection. In the late infection stage, a larger number of differentially transcribed genes were upregulated: genes involved in sulphate transport and sulphur metabolism, spermidine biosynthesis, fatty acid biosynthesis, pyoverdine biosynthesis, metabolism of specific amino acids (glycine, serine), transcription, protein folding, ribosomal proteins and RNA, taurine metabolism, phosphate transport, extracellular polymeric substance (EPS) biosynthesis, pyochelin biosynthesis, two-component systems, and antibiotic precursors (β-lactamase, isopenicillin N); and downregulated: genes associated with amino acids transport (branched amino acids, acidic amino acids), amino acids metabolism (tyrosine, glutamine, isoleucine/leucine, valine, histidine), carbon metabolism, lactate oxidation, oxidative phosphorylation, acetyl coenzyme A (acetyl-coA) metabolism, LPS synthesis, type IV pili synthesis, and virulence transcriptional regulators.

**Figure 5.**
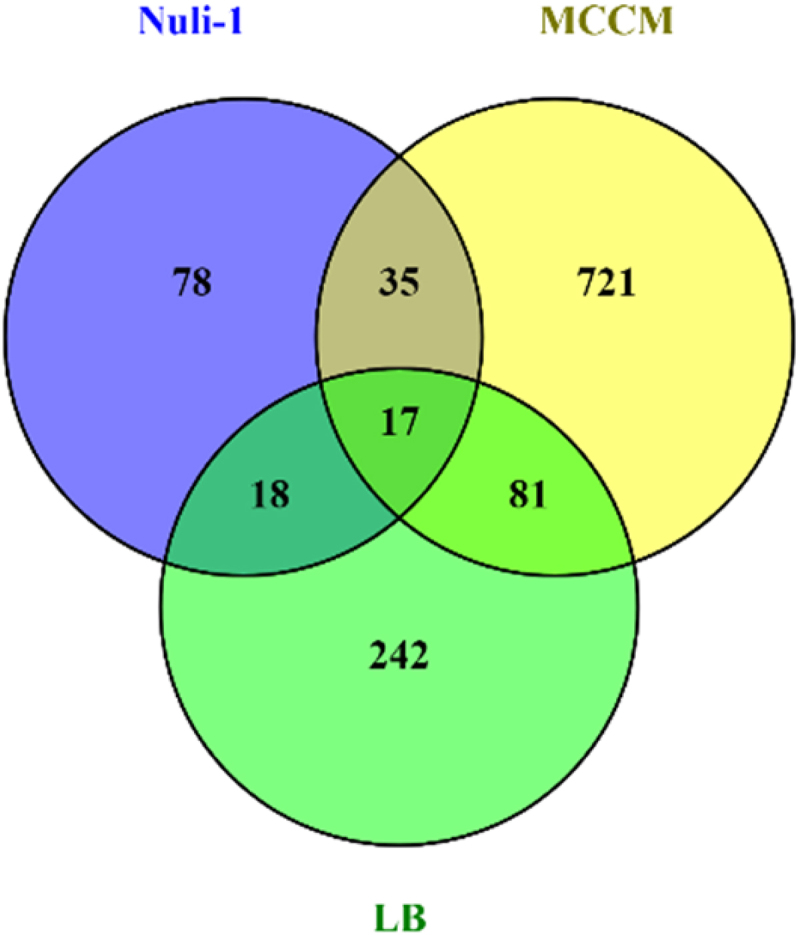
Venn diagram showing genes that are exclusively differentially transcribed in each media and the genes that are commonly expressed in each condition. For each condition, DEGs from 5 vs. 0min, 10 vs. 0min and 15 vs. 0min are included. The intersection of each condition represents the number of shared genes that are differentially expressed.

To understand if the transcriptional responses obtained from RNA-seq of samples acquired during lung epithelial cells exposure are specific to Nuli-1 cells growth condition or if they are caused only by the phage regardless of the bacterial growth conditions, the data was compared with the previously published bacterial transcriptomes in LB medium and MCCM medium [16]. To allow comparison, the previous data was re-analysed using the same bioinformatic tools and thresholds. In total, 17 genes were found to be differentially expressed in all growth conditions, regardless of the time point, 78 genes are specifically differentially transcribed in the Nuli-1 condition, 242 are specific for LB condition, and 721 for MCCM (Figure 5). Interestingly, among the 17 genes that are differentially expressed in all media, four are annotated as probable phage proteins, although their role during phage infection is unknown. Besides phage-related genes, genes involved in glycine cleavage, spermidine biosynthesis, post-translational modification (*tpbA*), tyrosine catabolism, glutathione metabolism, and leucine catabolism were also found to be commonly differentially transcribed in all growth conditions (Table S3). Genes exclusively differentially transcribed in the Nuli-1 condition can be seen in Table S4.

### Conserved induced phage responses between the different bacterial growth conditions

**Figure 6.**
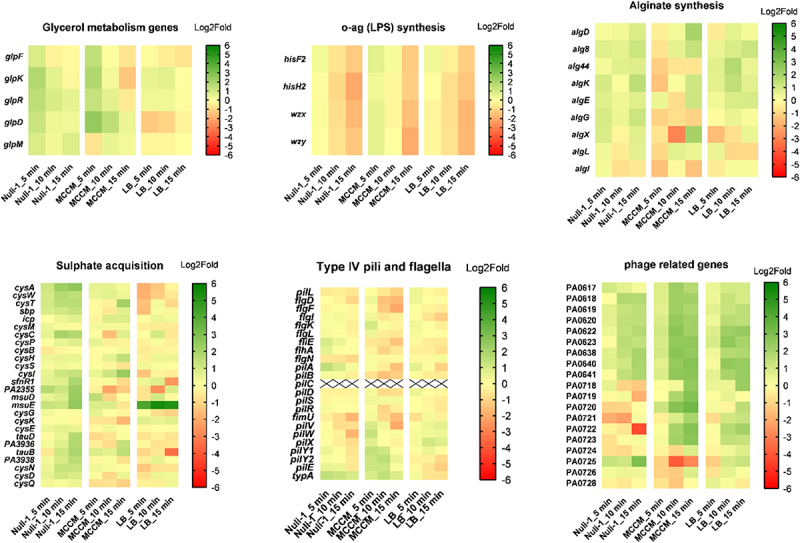
Representation of Log2Fold values for each categorical group of genes at different stages of infection (5-, 10-, and 15- vs. 0min) in bacteria adhered to Nuli-1 epithelial cells monolayer, grown on MCCM media, and grown on LB media. Log2Fold values are always relative to the expression level of uninfected bacteria at each respective growth condition. Plot was performed in GraphPad prism v8.0.1 with double gradient colourmap, using red for smallest value (Log2Fold = −6), yellow for baseline value (Log2Fold = 0), and green for largest value (Log2Fold = 6).

To understand the transcriptional changes that occurred in the different growth conditions during the phage infection, we combined previously obtained complementary data [16] with new data reported here and represented the Log2Fold values over time (5 vs. 0min, 10 vs. 0min, 15 vs. 0min) as a heat map (Figure 6). The first common transcriptional changes that appear to be triggered during phage infection regardless of the growth conditions are the genes related to spermidine biosynthesis. *spE2* is among the most significant differentially expressed genes. This gene is significantly upregulated in MCCM and Nuli-1 conditions and is downregulated in LB. Another consistently triggered transcriptional change was related with the increasing upregulation of hypothetical phage proteins and hypothetical prophage Pf1 genes in all bacterial growth conditions as phage infection progresses. On the other hand, the opposite is observed for the operon that comprises genes *hisF2, hisH2, wzx, wzy*. These genes are involved in the biosynthesis of the heteropolymer o-specific B-band O-antigen of LPS and are increasingly downregulated over time in all growth conditions. In addition, during the early stage of phage infection and specifically on bacteria adhered to lung epithelium and grown on MCCM, glycerol metabolism is induced, although upregulation of genes associated with this metabolism becomes less pronounced over time. Lastly, alginate-associated biosynthetic genes are also triggered after phage infection. Whereas the alginate-related genes are upregulated in the presence of LB medium and Nuli-1 epithelium, we observe a drastic shift from downregulation to upregulation in MCCM medium with the exception of gene *algI* (transcriptional regulator). The transcriptional profile for type IV pili and flagella-associated genes is less pronounced, although in general, these appear to be upregulated during early infection (for MCCM and Nuli-1 conditions) and subsequently become less transcribed over the course of infection.

In all growth conditions, *rsaL* (control of quorum sensing cascade) and *dpsI* (virulence related) genes also appear to be common phage targets, as they are downregulated over the course of infection, with the exception of *dpsI* in LB medium.

### Specific phage-induced responses in the presence of lung epithelial cells

**Figure 7.**
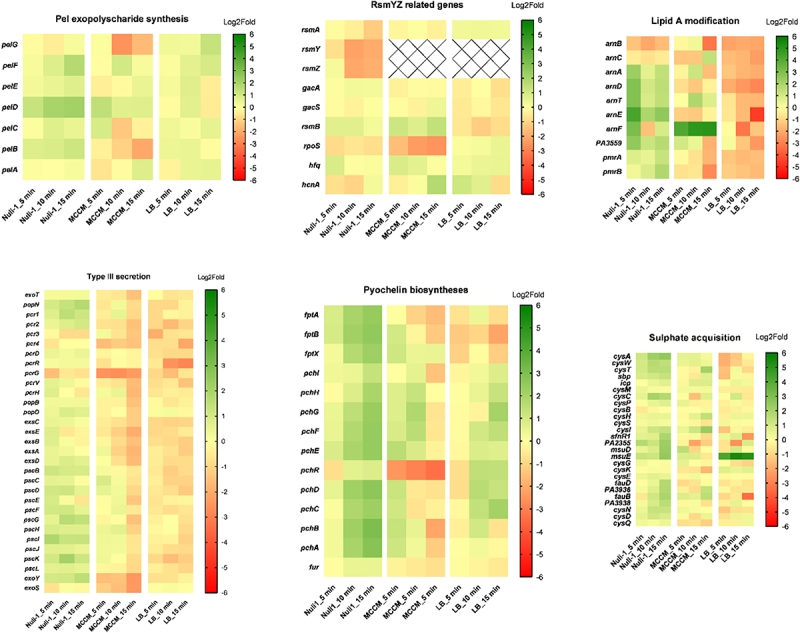
Representation of Log2Fold values for each group of genes that are specifically targeted at Nuli-1 at different stages of infection (5-, 10-, and 15- vs. 0min) in bacteria adhered to Nuli-1 epithelial cells monolayer, grown on MCCM media, and grown on LB media. Log2Fold values are always relative to the expression level of uninfected bacteria at each respective growth condition. Plot was performed in GraphPad Prism v8.0.1 with double gradient colourmap, using red for smallest value (Log2Fold = −6), yellow for baseline value (Log2Fold = 0), and green for largest value (Log2Fold = 6).

Even though the phage efficiently progresses with its infection and leads to major changes in the bacterial transcriptome, which seem to be common for all tested media, we observed specific transcriptional changes that were unique to the lung epithelial cell condition (Figure 7). Among these differences, the upregulation of sulphate-related genes over the infection time was observed only for the Nuli-1 condition, which indicates a requirement of this nutrient during phage infection, perhaps by providing a phage fitness advantage under this condition [41,42]. In addition, the transcription of the *arn* operon, which is associated with the modification of the lipid A component of LPS, is upregulated during infection when bacteria adhere to Nuli-1 epithelial cells. Likewise, the genes that positively regulate the *arn* operon, the PmrA/PmrB belonging to the two-component system, are also upregulated during infection.

Siderophore pyochelin synthesis-associated genes are also significantly differentially expressed in the Nuli-1 condition during phage infection, as well as in the LB medium, although slightly attenuated compared to the Nuli-1 environment. Considering the importance of iron homoeostasis in *P. aeruginosa*, the expression of genes involved in iron acquisition was evaluated in the three growth conditions (Figure S5). In the Nuli-1 condition, the overall set of iron-related genes do not seem to be differentially transcribed with the exception of pyochelin. By contrast, iron-related genes are generally upregulated in LB and a strong downregulation is observed in the MCCM medium, with the exception of *bfd* gene (bacterioferritin-associated ferredoxin). To better understand these differences between media, the transcription of the same genes at each medium without phage exposure was also examined. Comparison between the different conditions showed that bacteria are highly transcribing iron-related genes in the MCCM medium relative to the Nuli-1 and LB growth conditions, and the same genes are upregulated in the Nuli-1 condition compared to the LB medium. Therefore, bacteria are increasing expression of these genes in the following sequence: MCCM>Nuli-1>LB, indicating that the bacteria are showing the most iron starvation in MCCM, followed by the Nuli-1 condition.

Another interesting transcriptomic modulation during phage infection of bacteria adhered to lung epithelium is the consistent upregulation of genes involved in type III secretion, which in LB and MCCM seem to be downregulated during infection, relative to uninfected bacteria. Also, non-coding RNAs (ncRNAs) RsmY and RsmZ were found to be downregulated over the infection when bacteria adhere to Nuli-1 cells. The Log2Fold values of its transcriptional regulators, *gacA/gacS genes*, as well as *RsmYZ*’s final target (RsmA) and other genes such as *hfq* and polyribonucleotide nucleotidyltransferase encoding gene (*pnp)* involved in RsmYZ stability and degradation were also evaluated. These results demonstrated that *pnp* is upregulated during infection, especially in Nuli-1 and MCCM, and that *hfq* expression is reduced over the course of infection. The gene that encodes RsmA, a global virulence regulator, is exclusively downregulated in the Nuli-1 condition, although the genes that encode for its transcriptional regulators (*rsmY and rsmZ*), which are both downregulated throughout infection. On the other side, *gacA/gacS* genes seem to not be differentially expressed during infection, which is likely related to the non-transcription of *rsmY and rsmZ*. In addition, *pnp* which encodes a Polynucleotide Phosphorylase (PNPase) and is involved in the RsmY and RsmZ degradation, is upregulated in both MCCM and Nuli-1 conditions. This might explain the decay in *rsmYZ* genes transcripts since transcription of Hfq (involved in RsmYZ stabilization) is also decreasing over the infection, especially in Nuli-1 condition. As a final remark, another Nuli-1 specific transcriptional change was observed for the gene *mvaT*, which is increasingly downregulated over the infection.

## Discussion

**Figure 8.**
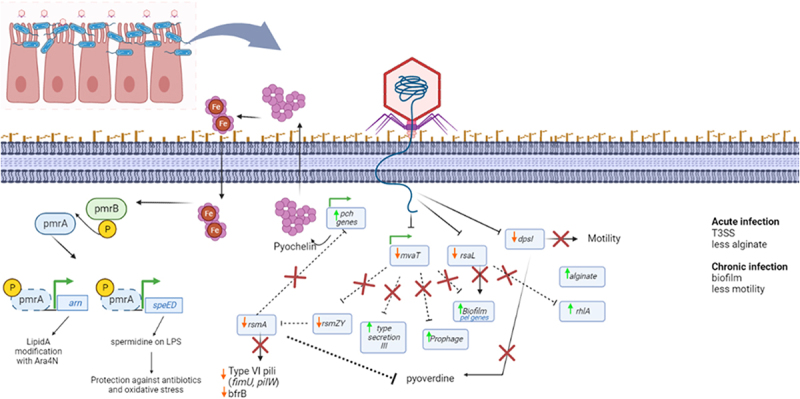
Main overview of transcriptional changes induced on bacteria by phage during infection in the presence of human lung epithelial cells.

Unveiling phage-bacteria interplay under conditions that mimic human physiology is a crucial step in phage biology research. Previously, we demonstrated that bacterial media conditions significantly impact the phage-bacteria transcriptome [16]. Here, we increased design complexity by tracking transcriptional changes under conditions that more closely mimic the human lung epithelium conditions. We observed that the phage was able to infect adhered bacteria and that it transcribes its genome following the same temporal gene transcription as revealed in other media (LB and MCCM medium). As previously observed, the bacterial growth rate appears to influence phage burst size and only a limited effect on phage transcription pace was detected. By contrast, significant differences in the bacterial transcriptome were observed (Figure 8), despite the lower sequencing depth obtained when combining phage/bacteria/human RNAs.

### Understanding the “Core” strategy of phage predation

In the presence of airway epithelial cells, LUZ19-induced transcriptional differences in the bacteria can be inferred to be part of a common predatory strategy. One of the most reported responses induced during *P. aeruginosa* phages infection is the upregulation of probable phage proteins and hypothetical prophage genes [7,8], which has been suggested to be a mechanism unleashed by the host stress response towards phage predation, leading to prophage escape. By using different growth conditions, our data confirmed that these results remain constant and indicate a conserved phage-bacteria interaction regardless of the growth environment.

From the main changes observed in the bacterial transcriptome during LUZ19 infection in lung epithelium presence, a general response on bacterial surface receptors concealment was observed, which was also observed in LB and MCCM growth conditions. For instance, the operon that comprises genes *hisF2, hisH2, wzx*, and *wzy*, involved in the biosynthesis of the heteropolymeric O-antigen (O-Ag) polysaccharide from LPS is commonly downregulated during infection, a phenomenon also observed during ϕKZ phage infection [17]. Hypothetically, this can be an approach for bacteria to acquire phage resistance or, a phage prompted action to prevent secondary infections [43]. In addition to inhibition of LPS synthesis, bacteria also express genes associated with alginate production during infection. If bacteria increase alginate production, this can ultimately result in bacterial receptor’s protection against phage infection. Nevertheless, alginate gene upregulation might be induced due to the stress imposed by phage infection on the cell since that alginate production is usually associated with higher bacterial tolerance to stress [44].

Consistently, the transcription of flagella and type IV pili were downregulated in all growth conditions, which has also been observed for several other *P. aeruginosa* phage infections. This downregulation is usually mediated by phage-proteins as a strategy to avoid bacterial superinfection and reduction of bacterial motility [18,45]. Therefore, regardless of bacterial physiological state, functions related to bacterial receptors concealment are promoted during phage predation, even under lung mimicking conditions. In the future, it might be important to understand if this induced bacterial surface modification is part of a phage-induced mechanism to avoid cell superinfection or a bacterial mechanism to resist to phage.

### Phage triggers alternative energy/substrates sources during infection in the presence of lung epithelium

Under more complex conditions, specific bacterial transcriptional differences can be observed. First, as previously reported on MCCM media, genes involved in glycerol metabolism are being specifically transcribed on phage early infection for this condition [16] but also under the presence of Nuli-1 epithelial cells. This metabolism is important during *P. aeruginosa* colonization of the airway sputum, as glycerol functions as an essential nutrient and appears to be involved as a precursor for virulence factor production [46]. Our data revealed that under lung mimicking conditions, the phage triggers this alternative pathway to acquire energy to attain optimal infection conditions. In addition, phage infection leads to a ‘sulphate starvation-like condition’. The usurpation of sulphate metabolism can be beneficial to the phage by increasing its fitness [42], especially when this substrate is in abundance, which seems to be the case under the presence of epithelial cells that can produce mucins, an important source of sulphate for *P. aeruginosa* under contact with host cells [47].

### Under lung physiology conditions, the phage employs hidden predation mechanisms by inducing LPS modification and pyochelin synthesis

A phage-prompted transcriptional response that was observed only in MCCM medium and under the presence of lung epithelial cells was the upregulation of spermidine biosynthesis. Spermidine is a polyamine pointed to be a very important substrate for efficient phage infection [48], playing a role in phage DNA stabilization and compaction by neutralizing the negatively charged phage genomes [49,50]. Indeed, polyamines seem to play an important role in the viral relationship between host cells and their metabolites, including viruses that infect bacteria, plants, and mammals [51]. Recently, polyamines have been identified as a new phage defence mechanism, where polyamines liberated from phage-lysed bacteria work as a danger signal to neighbour cells to prevent bacterial death mediated by phage, as this process is mediated by Gac/Rsm signalling. However, this mechanism might be counteracted by LUZ19-encoded proteins since the increase in polyamines does not lead to inhibition of LUZ19 phage replication/transcription, and according to our data genes that code for Gac/Rsm signalling are being downregulated during infection (compared to non-infected bacteria).

A distinct bacterial response was also captured specifically under the presence of epithelial cells, i.e., the upregulation of genes related to Lipid A modification with ara4N and the genes that code for its transcriptional regulators, *pmrA/pmrB*. Usually, the upregulation of this operon is related to LPS masking, to protect *P. aeruginosa* from the immune system or for the acquisition of antibiotic resistance against polymyxin B [52]. Curiously, PmrA/PmrB are also described to induce the expression of *speDE* genes as a response to low Mg^2+^ or the presence of extracellular DNA, protecting cells from oxidative damage and antibiotics by binding spermidine to LPS [53]. Therefore, under *in vivo* conditions, phages might activate additional mechanisms to protect the infected cell from other viruses’ attacks and probably, even the host immune system, as PmrA/PmrB sensing regulators are an important target under these conditions. This mechanism is usually triggered by PmrB when it senses an increase of periplasmatic iron or a low amount of Mg^2+^ and phosphorylates PmrA that in turn activates the expression of *arn* genes [54]. In addition, iron bounded to *Pseudomonas* quinolone signal (PQS) or siderophores on periplasm can also induce *arn* operon expression [53].

Considering that iron homoeostasis is critically important for *P. aeruginosa* survival, virulence, and pathogenicity [55,56], but also in the phage life cycle [57–59], and since our data shows strong upregulation of pyochelin siderophore under airway epithelial cells, we tracked the expression levels of iron-associated genes, although genes associated with iron acquisition (with exception to pyochelin) are poorly differentially expressed under these conditions. This is not the case in MCCM media nor in LB media, in which iron-associated genes are strongly downregulated and slightly upregulated over the infection, respectively. Pyochelin upregulation during phage infection was already observed during phage PAK_P4 infection as well as other iron acquisition associated genes including pyoverdine and haem acquisition associated genes [23]. This reveals that upon infection, iron metabolism (that is usually tightly regulated in bacteria) is completely deregulated, i.e. without phage exposure the bacteria are in iron-starvation (MCCM> Nuli-1> LB). Under phage exposure, it would be expected that genes associated with iron metabolism would be strongly upregulated because the production of new virions demands high iron pools that are incorporated in phage tails and enzyme production. Strangely this seems to occur oppositely, as genes are downregulated in the media that have a low amount of iron (MCCM) and slightly upregulated in LB (higher amount of iron). Surprisingly, in the Nuli-1 condition only pyochelin is significantly upregulated but not pyoverdine. Usually, these two siderophores are induced together under iron starvation. Furthermore, the siderophore that has a higher affinity to iron is pyoverdine [29,60], meaning that in more realistic conditions iron recruitment is not necessary during phage infection, as the upregulation of pyochelin is an unidentified mechanism with an unknown role during phage infection. This observation contradicts previous observations on standard bacterial growth media that usually report that iron is recruited for new viral particles production during phage predation and is crucial during the lytic cycle [58]. Lastly, an alternative iron acquisition mechanism that possibly contributes to the iron pool is the upregulation of Pf phage (it is upregulated in all growth conditions as referred before), since Pf was already described to function as an iron chelator [57]. During infection, this might be an alternative route to assure iron availability inside the cell instead of using standard mechanisms such as the expression of genes like pyoverdine. Nevertheless, the iron levels increase due to the Pf chelation-associated mechanism does not explain by itself the increasing expression of *pmrA/pmrB* and consequentially transcription activation of *arn* operon, which is specific to the presence of Nuli-1 epithelial cells. In the future, it would be worth studying the relationship between the expression of iron, spermidine, lipid A, and prophage associated genes during phage infection since they seem to be connected, especially in lung epithelial conditions.

### Shutdown of bacterial global virulence regulators might lead to successful phage infection under realistic conditions

Data from bacteria adhered to lung epithelial cells allow us to observe the downregulation of ncRNAs RsmY and RsmZ. These ncRNAs are responsible for sequestering of global virulence post-translational regulator, RsmA [61]. RsmA modulates type III secretion system (T3SS), type VI secretion system (T6SS), biofilm formation, quorum sensing (QS), and siderophores production [62,63]. The transcription of the *rsmA* gene, transcriptional regulators GacA/GacS (which control *rsmY* and *rsmZ* expression [64]), *pnp* gene (its product degrades RsmY and RsmZ [65,66]), and *hfq* (its product protects RsmY and RsmZ from PNPase degradation [63]) appear to be well correlated between the different growth conditions that were used. However, apart from other growth conditions, Nuli-1 epithelium data reveals that low levels of RsmYZ RNAs might be a consequence of its degradation by PNPase (upregulated) or by its transcription inhibition by PmrA/PmrB (upregulated) [67].

In addition, performing RNA-seq on infected bacteria adhered to epithelial cells enabled us to capture a significant downregulation of the *mvaT (P16)* and P15 genes, of which the former encodes a global virulence transcriptional regulator that binds to AT-rich sequences and silences genes [68–70]. MvaT is reported to repress the ncRNAs RsmY/RsmZ but is also involved in the repression of biofilm formation, T3SS, and prophage-associated genes [68–70]. By a specific mechanism that occurs only in lung epithelium, the phage strongly downregulates *mvaT*, which might result in an increase in T3SS-associated genes expression that we observed specifically in the Nuli-1 condition. T3SS is a virulence factor expressed by *P. aeruginosa* in acute infections to promote its pathogenicity by delivering a group of effectors in the host cell [71]. On one side, the T3SS system might be increasing bacterial pathogenicity and virulence towards cell exposure to both phage predation and *in vivo* conditions, on the other side, it can be a result of *mvaT* downregulation during infection. Indeed, bacterial MvaT was already described to be targeted the Mip protein, encoded by *Pseudomonas* phage LUZ24. The mentioned phage protein binds to MvaT and blocks it, impeding MvaT binding to AT-rich sequence portions on phage genomes, therefore avoiding MvaT-mediated silencing of phage genes [72].

In general, many of the significant changes observed in the Nuli-1 condition seem to be interlinked and described in *P. aeruginosa*, although these have not been connected and elucidated for phages. This justifies the importance of performing assays that use similar conditions to *in vivo* because under conventional LB medium these pathways might not be induced.

## Conclusions

In general, performing RNA-seq on phage-infected bacteria under complex growth conditions is attainable and proves that overall transcriptional interactions can be observed using these assays. This experimental design also highlights how adaptable the *P. aeruginosa* metabolism is, as the bacterium’s transcriptome is very distinct for the different growth conditions tested. In parallel, it becomes obvious how robust the hijacking mechanisms of the phage are, in terms of manipulating the bacterial host metabolism, despite critical differences at the onset of infection. However, the distinction between lab and real conditions influence in predatory mechanisms remains relevant and is likely phage-specific and should therefore be carefully considered in phage-bacteria interaction studies.

## Supplementary Material

Supplemental Material

## Data Availability

All data that were generated and analysed during this study are included in this article and its supplementary information files. The data that support the findings of this study are deposited into GEO database with an access number GSE213159.
