# Supplementary material for: Impact of phage predation on P. aeruginosa adhered to human airway epithelium: major transcriptomic changes in metabolism and virulence-associated genes
Source: RNA Biol. 2023 May 24;20(1):235–47. doi: 10.1080/15476286.2023.2216065 (PMC10215021; doi:10.1080/15476286.2023.2216065)
Supplement: Supplemental Material [file KRNB_A_2216065_SM2762.docx]

**Supplementary data**

**Table S1-** Absolute values of reads mapping for each sample of P. aeruginosa infected with LUZ19 phage under the presence of Nuli-1 epithelial cells. Samples that were sequenced a second time to increase the depth power, are highlighted in bold.

| **Sample ID** | **Total reads** | **Reads mapped to PAO1** | **Non-ribosomal reads mapped to PAO1** | **Reads mapped to LUZ19** | **Reads mapped to human** |
| --- | --- | --- | --- | --- | --- |
| t0 NuLi 1 | 22,655,247 | 1,164,875 | 587,178 | 44 | 21,394,902 |
| t0 NuLi 2 | 21,143,209 | 1,758,163 | 841,096 | 28 | 19,311,374 |
| t0 NuLi 3 | 25,952,330 | 2,400,326 | 1,113,004 | 47 | 23,442,263 |
| **t5 NuLi 1** | 34,036,980 | 1,143,479 | 589,164 | 71,559 | 32,712,664 |
| t5 NuLi 2 | 26,876,170 | 2,604,199 | 1,459,406 | 90,603 | 24,095,580 |
| **t5 NuLi 3** | 33,455,113 | 1,390,562 | 462,542 | 15,831 | 31,945,722 |
| t10 NuLi 1 | 23,001,713 | 1,285,799 | 846,118 | 139,799 | 22,695,025 |
| **t10 NuLi 2** | 32,195,544 | 1,278,064 | 797,544 | 375,499 | 30,448,997 |
| t10 NuLi 3 | 24,434,280 | 2,492,432 | 1,032,460 | 673,179 | 21,214,841 |
| t15 NuLi 1 | 18,940,027 | 929,769 | 664,378 | 849,826 | 17,127,628 |
| **t15 NuLi 2** | 34,363,784 | 1,114,105 | 560,916 | 5,737,561 | 27,420,602 |
| t15 NuLi 3 | 22,639,034 | 1,068,012 | 708,182 | 2,086,045 | 19,426,255 |

**
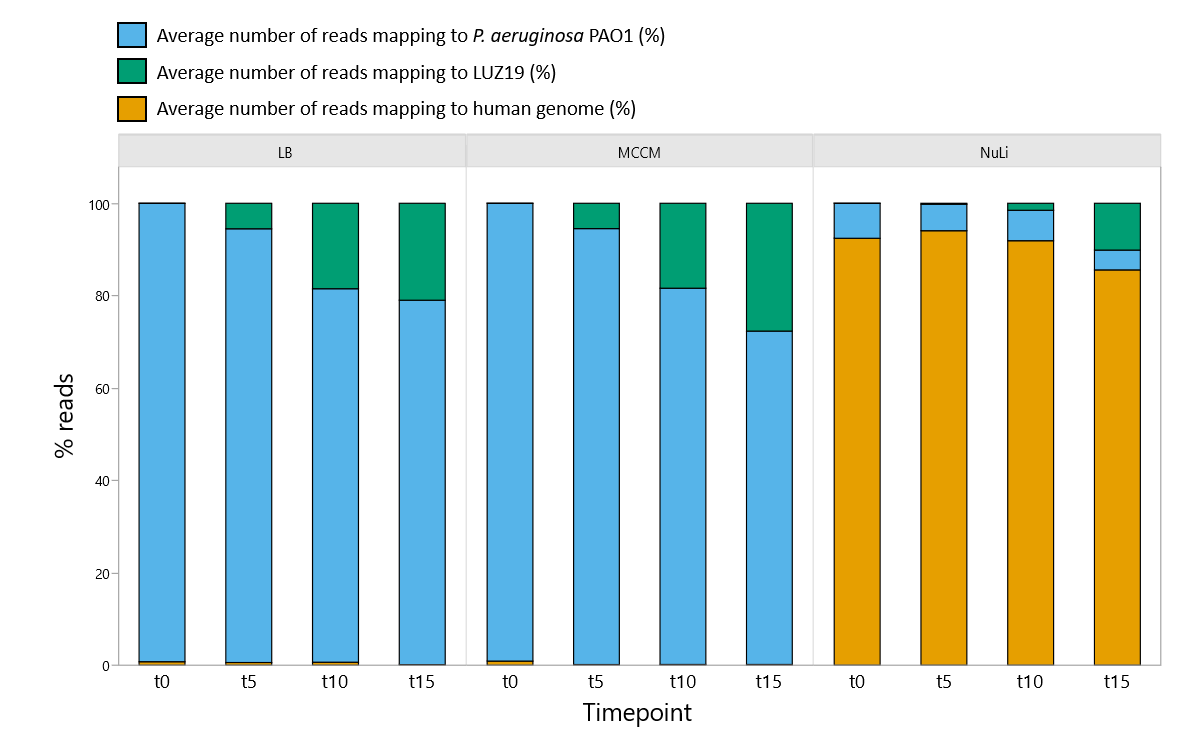
**

**Figure S1-** Percentage of reads mapping to P. aeruginosa PAO1 adhered to Nuli-1 epithelial cells and Pseudomonas LUZ19 phage in each sample acquired at 0 (control-uninfected bacteria), 5-, 10-, and 15-min post-infection. Blue bars represent the percentage of reads mapped to the P. aeruginosa PAO1 genome. Green bars represent the percentage of reads mapped to the LUZ19 phage, and orange bars represents the percentage of reads that map to the human genome.


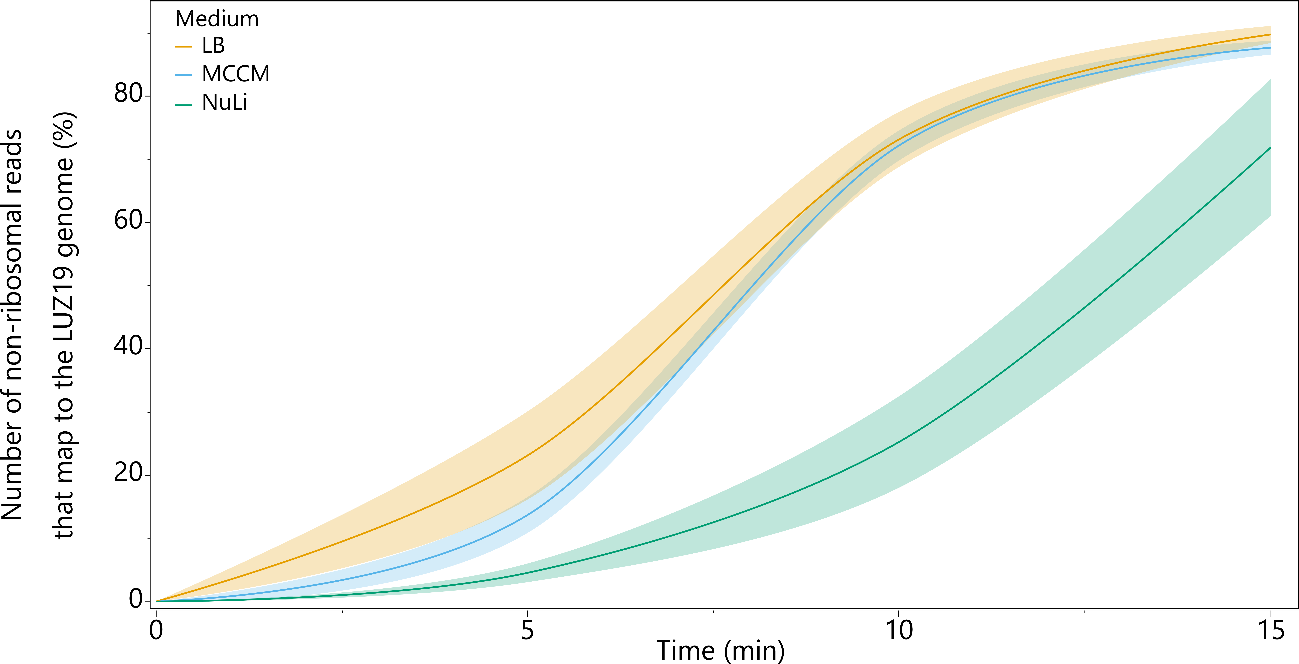


**Figure S2-** Percentage of RNA-sequencing reads that map to the LUZ19 genome over the time of infection of P. aeruginosa PAO1 grown in LB medium, MCCM, and Nuli-1 epithelial cells. This image shows the number of reads that maps to phage genome obtained in this study (under the presence of Nuli-1 epithelial cells) and previously obtained (in the presence of MCCM and LB medium), based on quantification in time points 0, 5, 10, and 15 min [16].


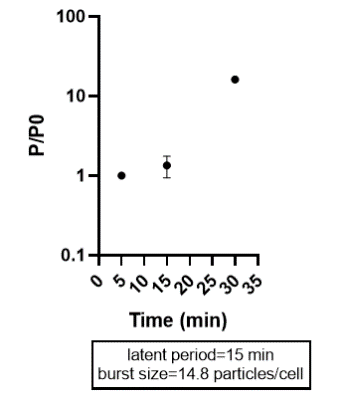


**Figure S3-** Pseudomonas LUZ19 one-step growth curve performed on P. aeruginosa PAO1 adhered to Nuli-1 epithelial cells monolayer.


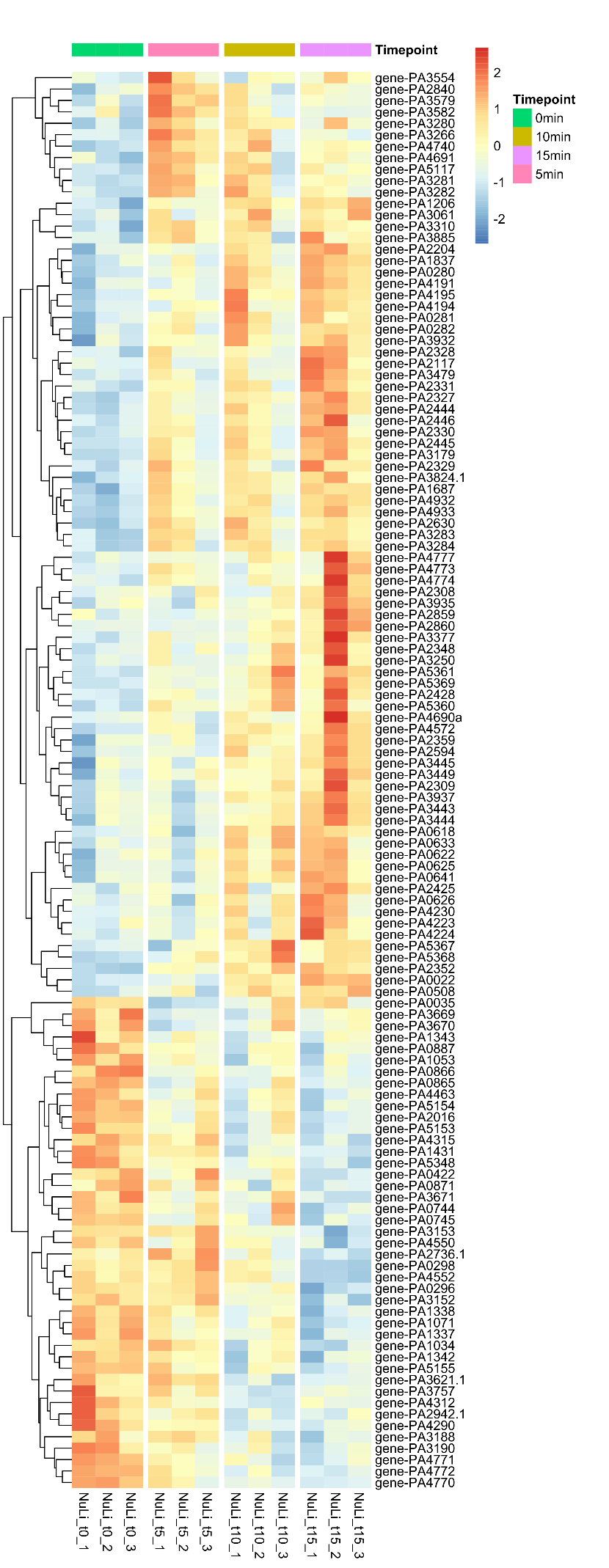


**Figure S4-** Heat map with normalized count values from each sample of Nuli-1 experiment considering DEGs from all time points (5 vs. 0min, 10 vs. 0min, and 15 vs. 0min respectively). Heat map was performed on using the pheatmap package in R.


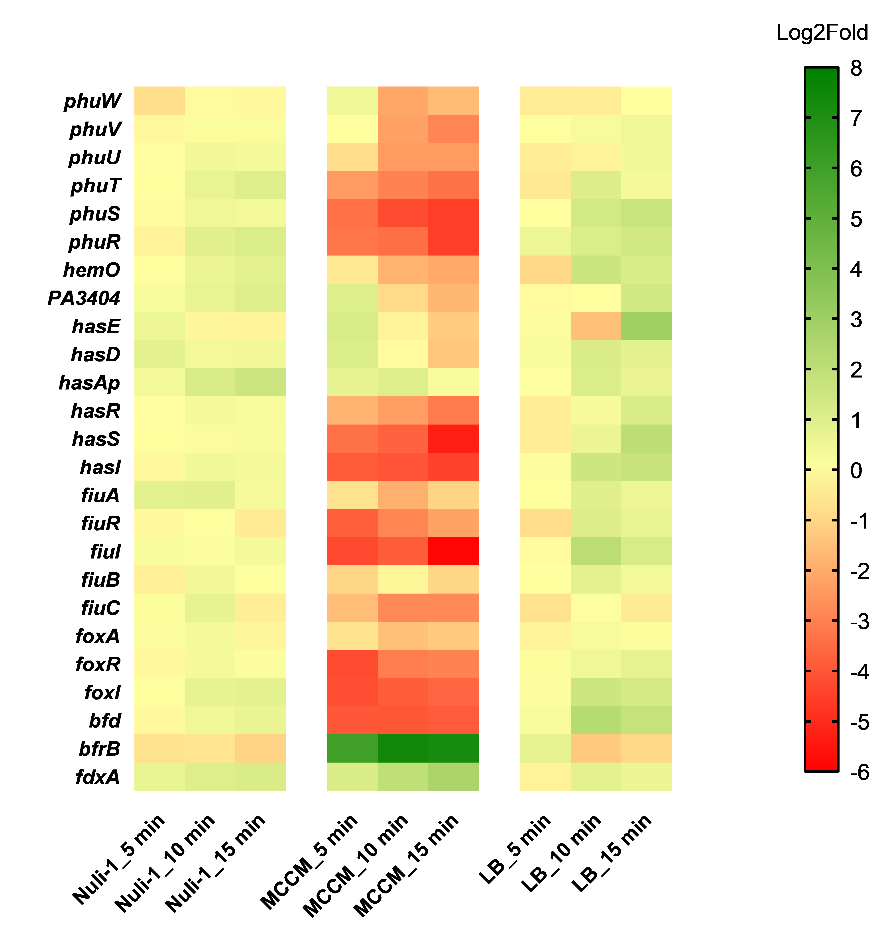


**Figure S5-** Representation of the expression of iron-related genes (Log2Fold values relative to uninfected bacteria) during each stage of phage infection, under the presence of different growth conditions. Plot was performed in GraphPad Prism v8.0.1 with double gradient colormap, using red for smallest value (Log2Fold = -6), yellow for baseline value (Log2fold=0), and green for largest value (Log2fold= 8).

**Table S2-** Complete group of DEG from P. aeruginosa PAO1 adhered to Nuli-1 epithelial cells monolayer during phage LUZ19 infection at different stages of infection using as control uninfected bacteria. Selection criteria of DEG were based on both Log2Fold ≥ |1.5| and padj≤0.05.

| **Locus Tag** | **Log2Fold** | **padj** | **Name** | **Biological role** |
| --- | --- | --- | --- | --- |
| Nuli-1 (5 vs. 0min) | | | | |
| PA0865 | -1,61 | 3,19E-03 | *hpd* | tyrosine catabolism |
| PA0866 | -1,63 | 4,07E-03 | *aroP2* | aromatic amino acid transport |
| PA2445 | 1,97 | 5,45E-03 | *gcvP2* | glycine cleavage system |
| PA3049 | -1,72 | 2,48E-03 | *rmf* | ribosome modulation factor |
| PA3266 | 1,95 | 5,32E-07 | *capB* | cold acclimation protein B |
| PA3280 | 2,64 | 2,21E-05 | *oprO* | Pyrophosphate-specific outer membrane porin OprO precursor |
| PA3554 | 2,31 | 2,30E-03 | *arnA* | beta-L-Ara4N-lipid A biosynthetic process |
| PA3582 | 1,80 | 5,45E-03 | *glpK* | glycerol metabolism |
| PA4541.3 | 1,53 | 4,06E-02 | tRNA-Asn | Aminoacyl-tRNA biosynthesis and tRNA charging |
| PA4740 | 1,52 | 1,31E-05 | *pnp* | prokaryotic degradosome |
| Nuli-1 (10 vs. 0min) | | | | |
| PA0280 | 2,24 | 1,79E-04 | *cysW* | sulfate transport |
| PA0281 | 1,67 | 1,98E-02 | *cysA* | sulfate transport |
| PA0866 | -1,75 | 2,08E-03 | *aroP2* | aromatic amino acid transport |
| PA1431 | -1,96 | 5,54E-04 | *rsaL* | regulatory protein |
| PA1687 | 1,70 | 1,45E-02 | *speE* | spermidine biosynthetic process |
| PA2445 | 2,24 | 3,00E-03 | *gcvP2* | glycine cleavage system |
| PA2942.1 | -2,10 | 7,45E-03 | P15 | non-coding RNA |
| PA3552 | -2,07 | 2,51E-03 | *arnB* | beta-L-Ara4N-lipid A biosynthetic process |
| PA3621.1 | -2,06 | 9,08E-04 | *rsmZ* | regulatory RNA |
| PA4225 | 1,79 | 4,67E-02 | *pchF* | Pyochelin biosynthesis |
| PA4226 | 1,97 | 1,52E-02 | *pchE* | Pyochelin biosynthesis |
| PA4230 | 2,23 | 1,31E-02 | *pchB* | Pyochelin biosynthesis |
| PA4231 | 2,01 | 2,55E-02 | *pchA* | Pyochelin biosynthesis |
| PA4315 | -1,58 | 6,23E-04 | *mvaT* | transcriptional regulator MvaT, P16 subunit |
| PA4572 | 1,59 | 1,20E-03 | *fklB* | protein folding |
| PA4771 | -1,63 | 3,79E-02 | *lldD* | L-lactate dehydrogenase |
| PA5170 | -1,88 | 3,85E-03 | *arcD* | arginine/ornithine antiporter |
| PA5360 | 1,89 | 2,25E-03 | *phoB* | two-component response regulator |
| PA5361 | 1,84 | 1,31E-02 | *phoR* | two-component response regulator |
| PA5367 | 1,67 | 1,46E-03 | *pstA* | phosphate transport |
| PA5368 | 2,09 | 1,75E-05 | *pstC* | phosphate transport |
| PA5369 | 2,35 | 3,61E-05 | *pstS* | phosphate transport |
| Nuli-1 (15 vs. 0min) | | | | |
| PA0280 | 2,56 | 2,4E-08 | *cysA* | sulfate transport |
| PA0282 | 1,61 | 4,8E-03 | *cysT* | sulfate transport |
| PA0296 | -1,76 | 1,2E-05 | *spuI* | Glutamylpolyamine syntheses |
| PA0298 | -1,57 | 1,4E-04 | *spuB* | Glutamylpolyamine syntheses |
| PA0527,1 | -1,98 | 5,8E-03 | *rsmY* | Regulatory RNA |
| PA0745 | -1,76 | 4,4E-03 | *dspI* | diffusible signal factor (DSF) CDA biosynthesis |
| PA0865 | -2,20 | 1,3E-10 | *hpd* | tyrosine catabolism |
| PA0866 | -2,14 | 2,7E-04 | *aroP2* | aromatic amino acid transport |
| PA0871 | -1,75 | 9,6E-03 | *phhB* | Phenylalanine metabolism |
| PA0887 | -1,74 | 6,6E-04 | *acsA* | acetyl-CoA biosynthetic process from acetate |
| PA1071 | -1,95 | 1,4E-04 | *braF* | branched-chain amino acid transport |
| PA1337 | -1,88 | 5,8E-05 | *ansB* | Glutamine metabolism |
| PA1338 | -1,97 | 6,0E-05 | *ggt* | Taurine and hypotaurine metabolism |
| PA1342 | -1,63 | 1,3E-03 | *aatJ* | Acidic amino acid transport |
| PA1431 | -2,40 | 9,0E-07 | *rsaL* | regulatory protein |
| PA1687 | 2,09 | 1,8E-04 | *speE* | spermidine biosynthetic process |
| PA1869 | 1,97 | 2,6E-02 | *acp1* | fatty acid synthesis |
| PA2000 | -2,57 | 9,5E-03 | *dhcB* | Valine, leucine and isoleucine degradation |
| PA2001 | -1,53 | 3,3E-02 | *atoB* | acetyl-CoA acetyltransferase |
| PA2015 | -1,58 | 6,4E-03 | *liuA* | leucine/isovalerate metabolism |
| PA2016 | -2,48 | 1,9E-03 | *liuR* | leucine/isovalerate metabolism |
| PA2356 | 2,37 | 4,3E-02 | *msuD* | sulfur metabolism |
| PA2425 | 1,50 | 1,7E-03 | *pvdG* | pyoverdine biosynthetic process |
| PA2444 | 3,07 | 1,8E-05 | *glyA2* | Glycine, serine and threonine metabolism |
| PA2445 | 3,14 | 2,0E-11 | *gcvP2* | glycine cleavage system |
| PA2446 | 3,00 | 2,8E-03 | *gcvH2* | glycine cleavage system |
| PA2637 | -1,52 | 2,3E-02 | *nuoA* | oxidative phosphorilation |
| PA2736.1 | -2,09 | 1,0E-02 | tRNA-Pro | Aminoacyl-tRNA biosynthesis and tRNA charging |
| PA2859 | 1,72 | 8,1E-03 | *greB* | transcription elongation facto |
| PA2942.1 | -1,86 | 2,3E-02 | P15 | non-coding RNA |
| PA3059 | 1,72 | 4,5E-02 | *pelF* | Extracellular polysaccharide biosynthesis |
| PA3061 | 2,15 | 8,7E-03 | *pelD* | Extracellular polysaccharide biosynthesis |
| PA3152 | -2,02 | 1,5E-04 | *hisH2* | histidine metabolism |
| PA3153 | -1,65 | 2,4E-03 | *wzx* | Lipopolysaccharide biosynthesis |
| PA3479 | 2,33 | 6,3E-03 | *rhlA* | rhamnolipid biosynthesis |
| PA3621.1 | -1,88 | 7,5E-04 | *rsmZ* | Regulatory RNA |
| PA3885 | 2,17 | 1,1E-02 | *tpbA* | protein tyrosine phosphatase |
| PA3935 | 1,75 | 8,6E-03 | *tauD* | Taurine metabolism |
| PA4191 | 2,02 | 5,3E-03 | isopenicillin-N synthase | isopenicillin N syntheses |
| PA4218 | 2,24 | 5,8E-03 | *ampP* | regulation of beta-lactamase activity |
| PA4219 | 2,24 | 7,5E-03 | *ampO* | regulation of beta-lactamase activity |
| PA4221 | 2,44 | 1,0E-03 | *fptA* | Fe(III)-pyochelin outer membrane receptor |
| PA4224 | 2,37 | 1,2E-03 | *pchF* | Pyochelin biosynthesis |
| PA4225 | 2,43 | 3,8E-04 | *pchG* | Pyochelin biosynthesis |
| PA4226 | 2,45 | 7,5E-04 | *pchE* | Pyochelin biosynthesis |
| PA4230 | 3,06 | 1,2E-04 | *pchB* | Pyochelin biosynthesis |
| PA4315 | -2,15 | 4,5E-07 | *mvaT* | transcriptional regulator MvaT, P16 subunit |
| PA4550 | -1,71 | 5,3E-03 | *fimU* | type IV pili assembly |
| PA4552 | -1,76 | 1,7E-03 | *pilW* | type IV pili assembly |
| PA4572 | 2,18 | 3,8E-05 | *fklB* | Protein folding |
| PA4690a | 1,72 | 1,8E-02 | 5S ribosomal RNA | |
| PA4770 | -1,82 | 5,2E-04 | *IldP* | Lactate oxidation |
| PA4771 | -2,01 | 6,8E-03 | *lldD* | Lactate oxidation |
| PA4773 | 2,11 | 3,5E-03 | *speD2* | spermidine biosynthetic process |
| PA4774 | 2,17 | 1,3E-03 | *speE2* | spermidine biosynthetic process |
| PA4777 | 1,84 | 2,7E-02 | *pmrB* | two-component regulator system |
| PA4932 | 1,56 | 1,7E-06 | *rplI* | 50S ribosomal protein L9 |
| PA5360 | 2,04 | 3,6E-03 | *phoB* | two-component response regulator |
| PA5361 | 1,80 | 4,2E-03 | *phoR* | two-component response regulator |
| PA5368 | 1,62 | 1,3E-04 | *pstC* | phosphate transport |
| PA5369 | 2,75 | 1,4E-07 | *pstS* | phosphate transport |
| PA5421 | -2,03 | 1,0E-02 | *fdhA* | Carbon metabolism |

**Table S3-** P. aeruginosa PAO1 genes that are differentially expressed in all bacterial growth conditions (LB medium, MCCM medium, and Nuli-1).

| **Locus tag** | **Gene name / Function** |
| --- | --- |
| PA0865 | ***hpd****:* 4-hydroxyphenylpyruvate dioxygenase |
| PA0625 | Probable bacteriophage protein |
| PA0633 | Hypothetical protein |
| PA4290 | Probable chemotaxis transducer |
| PA0622 | Probable bacteriophage protein |
| PA0626 | Hypothetical protein |
| PA0638 | Probable bacteriophage protein |
| PA0641 | Probable bacteriophage protein |
| PA1053 | Conserved hypothetical protein |
| PA1338 | ***ggt:*** Glutathione hydrolase large chain |
| PA2015 | ***liuA:*** Putative isovaleryl-CoA dehydrogenase |
| PA2204 | Putative binding protein component of ABC transporter |
| PA2446 | ***gcvH2:*** Glycine cleavage system H protein 1 |
| PA3187 | Probable ATP-binding component of ABC transporter |
| PA3235 | Conserved hypothetical protein |
| PA3885 | ***tpbA:*** Protein tyrosine phosphatase |
| PA4774 | ***speE2:*** Polyamine aminopropyltransferase 2 |

**Table S4-** P. aeruginosa PAO1 genes that are differentially expressed only in Nuli-1 condition, without the genes that encode for hypothetical proteins.

| **Locus tag** | **Gene name** | **Function** |
| --- | --- | --- |
| PA0022 | *tsaC* | Threonylcarbamoyl-AMP synthase |
| PA0282 | *cysT* | Sulfate transport system permease protein |
| PA0296 | *spuI* | Probable glutamine synthetase |
| PA0298 | *spuB* | Probable glutamine synthetase |
| PA0527.1 | *rsmY* | Regulatory RNA |
| PA0745 | [*dspI*](https://www.pseudomonas.com/feature/show?id=104241) | diffusible signal factor (DSF) in CDA biosynthesis |
| PA0871 | *phhB* | Pterin-4-alpha-carbinolamine dehydratase |
| PA1071 | *braF* | High-affinity branched-chain amino acid transport ATP-binding protein |
| PA1431 | *rsaL* | Regulatory protein RsaL |
| PA1869 | *acpP2* | Acyl carrier protein 2 |
| PA2000 | *dhcB* | dehydrocarnitine CoA transferase, subunit B |
| PA2001 | *atoB* | Acetyl-CoA acetyltransferase |
| PA2444 | *glyA2* | Serine hydroxymethyltransferase 2 |
| PA2736.1 | [tRNA-Pro](https://www.pseudomonas.com/feature/show?id=108260) | Aminoacyl-tRNA biosynthesis |
| PA2942.1 | P15 | ncRNA |
| PA3059 | *pelF* | extracellular polysaccharide biosynthesis |
| PA3061 | *pelD* | extracellular polysaccharide biosynthesis |
| PA3152 | *hisH2* | Imidazole glycerol phosphate synthase subunit HisH 2 |
| PA3280 | *oprO* | Pyrophosphate-specific outer membrane porin |
| PA3479 | *rhlA* | rhamnolipid surfactant biosynthesis |
| PA3621.1 | *rsmZ* | Regulatory RNA |
| PA3824.1 | tRNA-Leu | Aminoacyl-tRNA biosynthesis |
| PA3935 | *tauD* | Taurine dioxygenase |
| PA3936 | probable permease of ABC taurine transporter | |
| PA3937 | *tauB* | Taurine import ATP-binding protein |
| PA4191 | [isopenicillin-N synthase](https://www.pseudomonas.com/feature/show?id=111202) | |
| PA4219 | *ampP* | regulation of beta-lactamase activity |
| PA4230 | *pchB* | Pyochelin biosynthesis |
| PA4231 | *pchA* | Pyochelin biosynthesis |
| PA4315 | *mvaT* | Transcriptional regulator MvaT, P16 subunit |
| PA4541.3 | tRNA-Asn | |
| PA4552 | *pilW* | Type 4 fimbrial biogenesis protein |
| PA4690a | Paraquat-inducible protein A | |
| PA4770 | *lldP* | Lactate transport |
| PA4771 | *lldD* | Lactate conversion to pyruvate |
| PA4772 | probable ferredoxin | |
| PA4777 | *pmrB* | PmrB: two-component regulator system signal sensor kinase |
| PA5360 | *phoB* | Phosphate regulon transcriptional regulatory protein |
| PA5361 | *phoR* | Phosphate regulon sensor protein PhoR |
| PA5367 | *pstA* | Phosphate transport system permease protein |
| PA5368 | *pstC* | Membrane protein component of ABC phosphate transporter |
| PA5421 | *fdhA* | Glutathione-independent formaldehyde dehydrogenase |
